# Plasma non-esterified docosahexaenoic acid is the major pool supplying the brain

**DOI:** 10.1038/srep15791

**Published:** 2015-10-29

**Authors:** Chuck T. Chen, Alex P. Kitson, Kathryn E. Hopperton, Anthony F. Domenichiello, Marc-Olivier Trépanier, Lauren E. Lin, Leonardo Ermini, Martin Post, Frank Thies, Richard P. Bazinet

**Affiliations:** 1 Department of Nutritional Sciences, Faculty of Medicine, University of Toronto, FitzGerald Building, 150 College St., Room 306, Toronto, ON, Canada, M5S 3E2; 2 Program in Physiology and Experimental Medicine, Peter Gilgan Centre for Research and Learning, The Hospital for Sick Children, Toronto, ON, Canada, M5G 0A4; 3 School of Medicine and Dentistry, University of Aberdeen, Foresterhill, Aberdeen, UK

## Abstract

Despite being critical for normal brain function, the pools that supply docosahexaenoic acid (DHA) to the brain are not agreed upon. Using multiple kinetic models in free-living adult rats, we first demonstrate that DHA uptake from the plasma non-esterified fatty acid (NEFA) pool predicts brain uptake of DHA upon oral administration, which enters the plasma NEFA pool as well as multiple plasma esterified pools. The rate of DHA loss by the brain is similar to the uptake from the plasma NEFA pool. Furthermore, upon acute iv administration, although more radiolabeled lysophosphatidylcholine (LPC)-DHA enters the brain than NEFA-DHA, this is due to the longer plasma half-life and exposure to the brain. Direct comparison of the uptake rate of LPC-DHA and NEFA-DHA demonstrates that uptake of NEFA-DHA into the brain is 10-fold greater than LPC-DHA. In conclusion, plasma NEFA-DHA is the major plasma pool supplying the brain.

The brain has a unique fatty acid composition, being relatively enriched in certain saturated, monounsaturated and polyunsaturated fatty acids (PUFA), while very low in others. Within the brain, docosahexaenoic acid (DHA; 22:6n-3) is the major omega-3 (n-3) PUFA comprising about 10–15% of all fatty acids and over 50% of all brain PUFA^1^. DHA is a precursor to a series of bioactive molecules coined specialized pro-resolving mediators which includes the resolvins, protectins and maresins as well as docosahexaenoyl ethanolamide^2^,^3^. Collectively, DHA and its enzymatically synthesized products regulate numerous processes within the brain, including neurogenesis, cell survival, neuroinflammation and its resolution^2^,^4^,^5^,^6^,^7^,^8^,^9^. Altered DHA signaling has been implicated in a variety of neurological disorders and psychiatric diseases and is currently an active area of research for brain therapeutics^10^,^11^,^12^.

DHA cannot be synthesized *de novo* and must be obtained directly in the diet or via the conversion of shorter chain essential n-3 PUFA, including α-linolenic acid, to DHA predominately in the liver^13^,^14^. As the brain synthesis of DHA is very low, it is generally agreed that the brain is supplied with DHA from the plasma^15^. In adults, the concentration of DHA in the brain is at steady-state^8^,^16^, with the uptake rate closely approximating the consumption rate. Because of this, the rate of brain DHA uptake can be estimated from the rate of brain DHA loss.

In plasma, DHA is present in two major pools: 1) bound to albumin as non-esterified DHA (NEFA-DHA) or lysophosphatidylcholine-esterified DHA (LPC-DHA), or 2) in lipoproteins esterified to triacylglycerol (TAG), diacylglycerol (DAG), cholesteryl ester (CE), phospholipids or other lysophospholipids. There has been considerable controversy as to which plasma fatty acid pools supply the brain with DHA. DHA within lipoproteins, NEFA-DHA and LPC-DHA have all been proposed not only to enter the brain, but to be the major pool supplying the brain with DHA^17^. Plasma lipoproteins account for the largest plasma pool of DHA; however, lipoprotein receptors do not transport cholesterol into the brain and LDL and VLDL receptor knockouts demonstrated that these receptors are not necessary for maintaining brain DHA levels^17^,^18^. With regards to the plasma NEFA-DHA pool, the observation that the rate of NEFA-DHA uptake into the brain closely matches the rate of brain DHA consumption support the hypothesis that plasma NEFA-DHA is the major source of DHA for the brain^13^,^16^. However, there is also evidence that LPC-DHA can supply DHA for the brain, as radioactive brain DHA levels are higher upon intravenous (iv) infusion of radiolabeled LPC-DHA compared to NEFA-DHA in developing rats, where DHA accretion is substantial^19^,^20^,^21^. This finding was recently confirmed in adult mice and interpreted as LPC-DHA being the major plasma pool supplying the brain with DHA^22^. However, this conclusion suggests that much more DHA is entering the brain than is being consumed, which is inconsistent with the finding that there is minimal, if any, accretion of DHA in the adult brain^16^. Therefore, to test the contribution of various DHA containing plasma pools to brain DHA uptake we used a series of kinetic approaches that account for the amount of exposure of the brain to the radiotracer and the specific activity of the plasma lipid pool^23^,^24^. We measured the uptake rate of iv infused NEFA-DHA into the brain, brain DHA uptake upon oral administration which could occur from multiple plasma pools, and the rate of brain DHA consumption. After accounting for an experimentally derived brain DHA uptake rate from NEFA-DHA, the brain uptake from esterified DHA (including LPC-DHA) was negligible, and uptake from NEFA-DHA was similar to the brain DHA consumption rate. We then reproduced the finding that iv infusion of radiolabeled LPC-DHA leads to higher brain radioactivity compared to NEFA-DHA; however, upon correcting for brain exposure and/or the plasma pool size, NEFA-DHA was determined to be the major plasma pool supplying the brain. In our last experiment, we continuously infused NEFA-DHA and LPC-DHA and found that the uptake rate of plasma NEFA-DHA into the brain was 10-fold higher than LPC-DHA.

## Results

### Brain DHA concentration and distribution

Similar to other reports, the brain total phospholipid DHA concentration of rats fed a chow containing 5% α-linolenate as the only n-3 PUFA was 5956 ± 477 nmol/g brain (Supplementary Fig. 1a)^25^. Upon further separation of the major structural phospholipid classes, DHA was highly enriched in ethanolamine glycerophospholipids (EtnGpl) (4826 ± 558 nmol/g brain) as compared to choline glycerophospholipids (ChoGpl) (852 ± 57 nmol/g brain); whereas between the major signaling phospholipids, DHA was found mostly esterified to phosphatidylserine (PtdSer) (1676 ± 302 nmol/g brain) as compared to phosphatidylinositol (PtdIns) (75 ± 18 nmol/g brain). DHA-containing phospholipid species were ubiquitous across brain regions (Supplementary Fig. 1b,c).

### Brain DHA uptake

**Figure 1:**
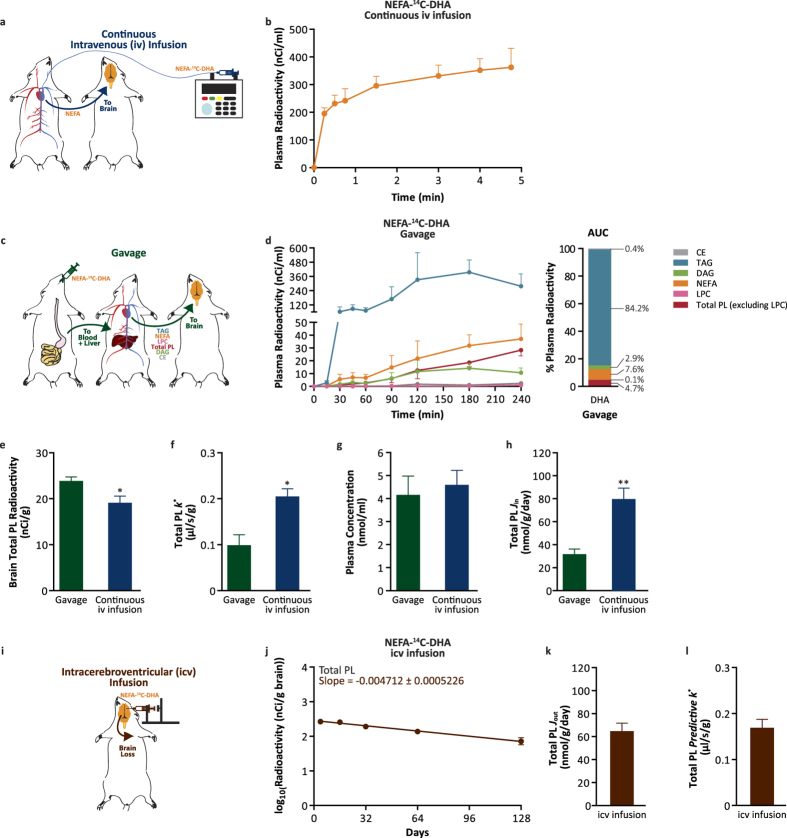
NEFA-DHA uptake by the brain. (**a**) Schematic diagram of kinetic modeling following continuous intravenous (iv) infusion. (**b**) Plasma lipid radioactivity upon continuous iv infusion of 86 μCi NEFA-^14^C-DHA over 5 minutes (n = 6). Radioactivity from other plasma lipid pools was below detection, 23 pCi. (**c**) Schematic diagram of kinetic modeling following NEFA-^14^C-DHA gavage. (**d**) Plasma lipid radioactivity upon NEFA-^14^C-DHA oral gavage over 4 hours (n = 4) and the percent composition of total radiolabeled lipid pools in the plasma post-gavage of NEFA-^14^C-DHA. (**e**) Brain radioactivity of total phospholipids from rats gavaged or continuously iv infused with NEFA-^14^C-DHA. (**f**) The uptake incorporation coefficient of NEFA-^14^C-DHA (k^*^) into brain total phospholipids from rat gavage or continuous iv infusion. (**g**) The plasma concentration of NEFA-DHA prior to NEFA-^14^C-DHA gavage or continuous iv infusion. (**h**) The net incorporation rate of DHA (*J*_in_) to brain total phospholipids from rats gavaged or iv infused with NEFA-^14^C-DHA. (**i**) Schematic diagram of kinetic modeling following intracerebroventricular (icv) infusion. (**j**) Brain loss curve of esterified ^14^C-DHA from brain total phospholipids including lysophosphatidylcholine over 128 days (n = 3–4 independent samples per time point). (**k**) The loss of DHA from brain total phospholipids (rate of loss, *J*_out_) from rats infused icv with NEFA-^14^C-DHA which was not significantly different from the *J*_in_ of rats continuously iv infused with NEFA-^14^C-DHA. (**l**) Calculated predictive k^*^ from the NEFA-^14^C-DHA icv infusion which was not significantly different from k^*^ from NEFA-^14^C-DHA iv infusion. All data are expressed as mean ± SEM; whereas percent plasma radioactivity data are expressed as mean. ^*^P < 0.05 and ^**^P < 0.01 indicate significant difference from gavage using Students’ *t*-test. AUC, area under the curve; CE, cholesteryl ester; DAG, diacylglycerol; LPC, lysophosphatidylcholine; NEFA, non-esterified fatty acid; PL, phospholipids; TAG, triacylglycerol.

To examine the rate of uptake of NEFA-DHA into the brain, albumin-bound NEFA-^14^C-DHA was continuously infused into the jugular vein of rats over a 5-minute period while blood samples were collected from the carotid artery (Fig. 1a). The 5-minute continuous infusion captured the appearance of NEFA-^14^C-DHA in the plasma (Fig. 1b). Measures for the uptake coefficient (k^*^) and rate of NEFA-DHA incorporation (*J*_in_) were 0.20 ± 0.02 μl/s/g and 80 ± 10 nmol/g/day, respectively (Eqs 4 and 5). To determine the relative contribution of esterified and NEFA-DHA to total brain DHA uptake, NEFA-^14^C-DHA was gavaged (Fig. 1c) to rats resulting in the appearance of ^14^C-DHA in several plasma esterified pools as well as the plasma NEFA pool (Fig. 1d; Supplementary Fig. 2 for confirmation of tracer identity. Similar to others, we observed that DHA made up greater than 99% of the radioactivity^16^). By continuous sampling from the carotid artery, it was determined that ^14^C-DHA appeared largely in triacylglycerol (TAG; 84% of total plasma radiotracer) and relatively lower amounts were found esterified in phospholipids (total phospholipids excluding LPC; 4.7%), diacylglycerol (DAG; 2.9%), cholesteryl esters (CE; 0.4%) and lysophosphatidylcholine (LPC; 0.13%). NEFA-^14^C-DHA was the second most abundant radiolabeled lipid pool in the plasma after NEFA-^14^C-DHA gavage accounting for 7.6% of total plasma radiotracer area under the curve (Fig. 1d). Radiolabeled LPC-DHA did not appear in the plasma until 3 hours post-ingestion of NEFA-^14^C-DHA which is consistent with it being secreted from the liver and not upon intestinal absorption (Fig. 1d). Using the measured k^*^ for NEFA-DHA (Fig. 1f), it was determined that the uptake of esterified DHA (k^*^_ES_) was −0.0089 ± 0.002 μl/s/g suggesting that esterified DHA including LPC-DHA is not, quantitatively, a major source for supplying the brain with DHA (Equations 1, 2, 3; Supplementary Table 1 for individual phospholipid species). Therefore, if it is assumed that only the radiolabeled DHA in the plasma NEFA pool post-gavage enters the brain, this leads to a k^*^ of 0.098 ± 0.05 μl/s/g and a net rate of incorporation (*J*_in_) of 32 ± 9 nmol/g/day (Fig. 1f,h Eqs 4 and 5). Collectively, we find that the k^*^ and *J*_in_ upon a 5-min continuous infusion are slightly higher than would be predicted from the plasma NEFA pool upon oral gavage of the NEFA-^14^C-DHA tracer (Fig. 1f,g). One reason for this might be error in estimating the AUC between the time points over a 4 hour study and due to acute catabolism of the tracer upon its entry into the brain during the 4 hours which has been estimated to be as much as 20% for PUFA^23^. While the mechanism for the decrease in PUFA radioactivity is not clear, it is possible that a proportion of the tracer enters an unstable pool within the brain.

To examine whether these rates of brain DHA uptake were similar to rates of DHA loss, the rate of loss of DHA from brain phospholipids was determined by administering NEFA-^14^C-DHA intracerebroventricularly and calculating the slope of DHA loss from total phospholipids (Fig. 1i, Supplementary Fig. 3 for individual phospholipid species). This measured loss half-life (*t*_1/2_) of 64 ± 7 days (Equation 6) translated to rate of loss from brain phospholipids (*J*_out_) of 65 ± 7 nmol/g/day (Fig. 1k, Equation 7) and a predictive k^*^ of 0.17 ± 0.02 μl/s/g (Fig. 1l, Equation 8). The *t*_1/2_ and *J*_out_ are in broad agreement with previous reports by DeMar *et al*. and Lin *et al*., with minor discrepancies possibly related to differences to animal ages, diets and strain^16^,^26^. Therefore, it is important that comparisons of fatty acid kinetic parameters from various models be from studies conducted under similar conditions. The k^*^ and *J*_in_ from the continuous iv infusion are not significantly different from the predictive k^*^ and *J*_out_ again suggesting that plasma NEFA-DHA can replace brain DHA loss to maintain homeostatic levels of brain DHA. The results predict that plasma NEFA-DHA is the major contributor to brain DHA *in vivo*.

### The relative partitioning of LPC-DHA into the brain compared with NEFA-DHA

**Figure 2:**
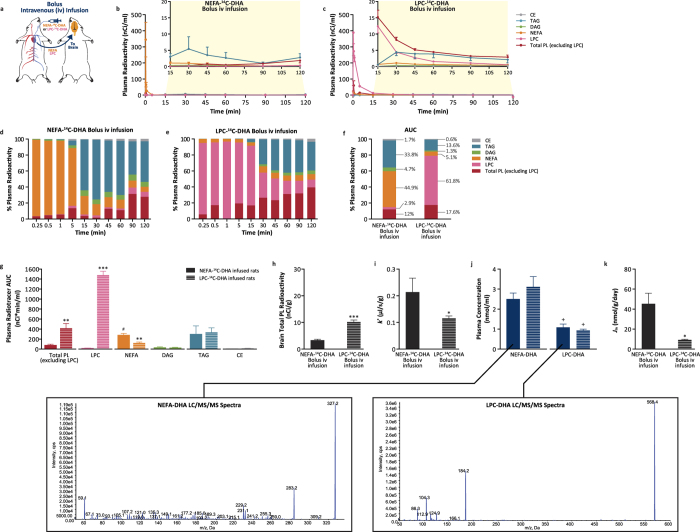
Plasma LPC-DHA contribution to brain total DHA uptake via bolus iv infusion kinetic modeling. (**a**) Schematic diagram of iv infusion kinetic modeling. (**b**,**c**) Plasma lipid radioactivity from NEFA-^14^C-DHA or LPC-^14^C-DHA bolus iv infusion (13 μCi) followed by 2-hour plasma sampling (n = 4 each). (**d,e**) Percent composition of various radiolabeled lipid pools in the plasma at each time point following an acute iv bolus infusion of NEFA-^14^C-DHA or LPC-^14^C-DHA. (**f**) Percent composition of various radiolabeled lipid pools in the plasma over 2-hour period following an acute iv bolus infusion of NEFA-^14^C-DHA or LPC-^14^C-DHA. (**g**) Plasma radiotracer exposure from various lipid pools after bolus iv infusion of NEFA-^14^C-DHA or LPC-^14^C-DHA. (**h**) Brain radioactivity of total phospholipids from rats iv infused with bolus NEFA-^14^C-DHA or LPC-^14^C-DHA. (**i**) The uptake incorporation coefficient (k^*^) into brain total phospholipids from rats iv infused with bolus NEFA-^14^C-DHA or LPC-^14^C-DHA. (**j**) The baseline plasma concentration of total NEFA-DHA and LPC-DHA prior to NEFA-^14^C-DHA or LPC-^14^C-DHA bolus iv infusion. LC/MS/MS spectra of NEFA-DHA and LPC-DHA. (**k**) The net incorporation rate of DHA (*J*_in_) into brain total phospholipids from rats iv infused with bolus NEFA-^14^C-DHA or LPC-^14^C-DHA. Percent plasma radioactivity data are expressed as mean. Remaining data are expressed as mean ± SEM. ^*^P < 0.05, ^**^P < 0.01 and ^***^P < 0.001 indicate significant difference from NEFA-^14^C-DHA infused rats. ^+^*P* < 0.01 indicates significant difference between plasma LPC-DHA and NEFA-DHA concentration. ^#^*P* < 0.001 indicates significant difference between radiolabeled LPC pool from LPC-^14^C-DHA infused rats and radiolabeled NEFA-DHA pool from NEFA-^14^C-DHA infused rats. Students’ *t*-test was used to determine significant difference. See Fig. 2 for abbreviations.

Earlier work demonstrated that upon iv infusion of a bolus of radiolabeled NEFA-DHA or LPC-DHA, that LPC-DHA preferentially targeted the brain^19^,^20^. Recently, with the discovery of a putative LPC-DHA transporter, and using similar experiments, it was concluded that LPC-DHA represents the major mechanism by which DHA enters the brain^22^. However, the results presented above (Fig. 1), are not compatible with the hypothesis that LPC-DHA is the major plasma pool supplying the brain. Thus, we set out to reproduce the finding of higher brain radioactivity following iv LPC-^14^C-DHA infusion. This time, however, the amount of DHA exposure to the brain was measured, which was not considered in the previous report. Similar to other reports^19^,^22^, a bolus iv infusion of radiolabeled NEFA-DHA or LPC-DHA was used and brains were collected 2 hours post-infusion (Fig. 2a). Consistent with the short half-life of plasma NEFA of approximately 30 seconds, NEFA-^14^C-DHA decreased 207-fold from the peak initial level within 5 minutes (Fig. 2b). In contrast, radiolabeled LPC-DHA was still elevated at 5 minutes and had only decreased 7-fold from the peak. Furthermore, at 30 minutes, the reduction in plasma LPC-^14^C-DHA levels was merely 88-fold as compared to a 339-fold reduction in plasma NEFA-^14^C-DHA levels, suggesting a much longer half-life for LPC-DHA (Fig. 2c). This translated to higher total relative LPC-^14^C-DHA exposure (61.8% of total plasma radiotracer) in rats receiving LPC-^14^C-DHA as compared to NEFA-^14^C-DHA exposure (44.9% of total plasma radiotracer) in rats receiving NEFA-^14^C-DHA (Fig. 2d–f). Collectively, the AUC for NEFA-DHA in the iv NEFA-^14^C-DHA infused rats was 282 ± 32 nCi*min/ml, while the AUC for plasma LPC-DHA in the iv LPC-^14^C-DHA infused rats was 1478 ± 80 nCi*min/ml (Fig. 2g). Similar to other reports, we found that radiolabeled brain DHA was 3-fold higher in iv LPC-^14^C-DHA infused animals compared those receiving NEFA-^14^C-DHA (Fig. 2h). However, because the radiolabel appeared in multiple plasma pools over the 2 hours, it is unclear which plasma pool would have supplied the brain with DHA. Nevertheless, we corrected brain radioactivity for either plasma LPC-^14^C-DHA or NEFA-^14^C-DHA exposure, which was 5.2 fold higher for LPC-DHA than NEFA-DHA (Fig. 2g). An uptake coefficient (k^*^) can be calculated, which was significantly higher for NEFA-DHA (0.21 ± 0.05 μl/s/g) than LPC-DHA (0.12 ± 0.009 μl/s/g) (Fig. 2i). Finally, to determine the rate of brain DHA uptake, the concentration of plasma NEFA-DHA and LPC-DHA was measured using LC/MS/MS and found that the concentration of LPC-DHA was 64% lower than NEFA-DHA (Fig. 2j). Applying the plasma concentration to the k^*^ for LPC-DHA and NEFA-DHA, the net rate of entry (*J*_in_) into the brain from the plasma NEFA pool (46 ± 10 nmol/g/day) is 5-fold higher than the rate of entry from the plasma LPC-DHA pool (9 ± 0.6 nmol/g/day) (Fig. 2k). Because a small fraction of LPC-DHA is in the form of lipoproteins, this method slightly overestimates albumin-bound plasma LPC-DHA. However, similar to the gavage experiment, if other pools are entering the brain, these rates could be overestimates. Since the k^*^ from the current bolus iv NEFA-DHA infusion study is similar to our results with continuous iv NEFA infusion, it suggests that, at least for this pool, it is not an overestimate.

**Figure 3:**
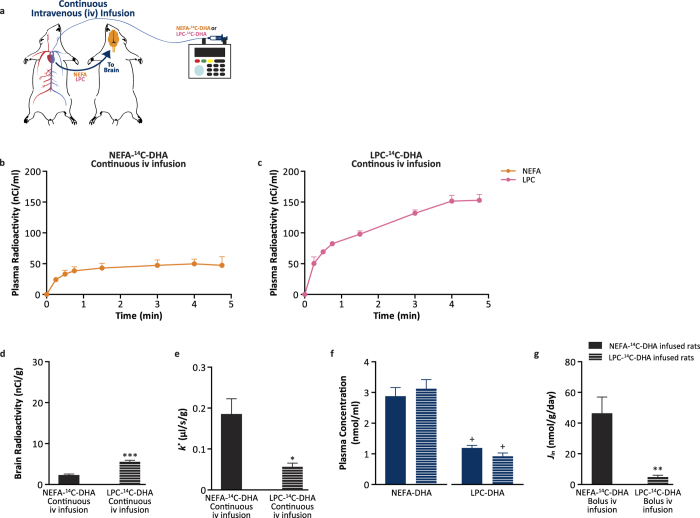
Plasma LPC-DHA and NEFA-DHA contribution to brain total DHA uptake via continuous iv infusion kinetic model. (**a**) Schematic diagram of continuous iv infusion kinetic modeling. (**b,c**) Plasma curve from continuous iv infusion of low-dose (10 μCi) NEFA-^14^C-DHA or LPC-^14^C-DHA over 5 minutes (n = 4 each). (**d**) Brain radioactivity of total phospholipids from rats iv infused with NEFA-^14^C-DHA or LPC-^14^C-DHA. (**e**) The uptake incorporation coefficient (k^*^) into brain total phospholipids from rats continuously iv infused with NEFA-^14^C-DHA or LPC-^14^C-DHA. (**f**) The baseline plasma concentration of total NEFA-DHA and LPC-DHA prior to NEFA-^14^C-DHA or LPC-^14^C-DHA continuous iv infusion. (**g**) The net incorporation rate of DHA (*J*_in_) into brain total phospholipids from rats continuously iv infused with NEFA-^14^C-DHA or LPC-^14^C-DHA. All data are expressed as mean ± SEM. ^*^P < 0.05, ^**^P < 0.01 and ^***^P < 0.001 indicate significant difference from NEFA-^14^C-DHA infused rats using Students’ *t*-test. ^+^*P* < 0.01 indicates significant difference between plasma LPC-DHA and NEFA-DHA concentration. See Fig. 2 for abbreviations.

To circumvent the repackaging of infused radiotracer into multiple plasma pools and to control for exposure, the uptake of NEFA-^14^C-DHA and LPC-^14^C-DHA was quantified using the continuous 5-minute infusion model (Fig. 3a). Consistent with the shorter half-life of NEFA-DHA, the plasma radioactivity appeared to plateau around 1 min (Fig. 3b), while plasma radioactivity increased during the study for the LPC-DHA (Fig. 3c). At the 5-minute time point, 97% of the total lipid radioactivity in the ^14^C-DHA-infused rats was in the plasma NEFA pool and 97% of the total lipid radioactivity in LPC-^14^C-DHA-infused rats was in the plasma phospholipid pool. As predicted from the previous experiments, brain radioactivity was significantly higher upon 5-minute LPC-^14^C-DHA infusion (Fig. 3d). After correction for plasma exposure, however, the incorporation coefficient (k^*^) was significantly higher in the group receiving NEFA-^14^C-DHA (0.18 ± 0.04 μl/s/g) than LPC-^14^C-DHA (0.06 ± 0.009 μl/s/g) (Fig. 3e). The elevated k^*^ in the ^14^C-DHA-infused rats was magnified upon correction for the plasma pool sizes (Fig. 3f) as plasma NEFA-DHA pool (*J*_in_ = 46 ± 11 nmol/g/day) contributes 10-fold more DHA than plasma LPC-DHA (*J*_in_ = 4.7 ± 1.2 nmol/g/day) (Fig. 3g). Importantly plasma concentrations of NEFA-DHA and LPC-DHA is this and the previous experiments are similar to the normal physiological range reported in the literature^27^,^28^. The replacement half-life of DHA in brain total phospholipids would be 65 ± 12 days from the plasma NEFA-DHA pool and 676 ± 122 days from the plasma LPC-DHA pool (calculated based on the concentrations from Supplementary Fig. 1). Furthermore, if use the experimentally derived k* (Fig. 3e) for LPC-DHA to predict the amount of brain radioactive DHA upon the oral gavage of NEFA-^14^C-DHA (Fig. 1e), this leads to a 148 fold underestimate of the measured radioactivity. The incorporation coefficients and net rate of entry for NEFA-DHA were consistent across all studies for the plasma NEFA-DHA, despite differences in time and dose. This is not surprising as uptake of plasma NEFA-DHA is thought to be via passive diffusion and the models used in this study are time-independent^29^,^30^,^31^,^32^,^33^.

## Discussion

Using several kinetic models, these studies demonstrated that plasma NEFA-DHA is the major plasma pool supplying the brain with DHA in free-living adult rats. The finding that plasma NEFA-DHA is the major plasma pool supplying the brain does not negate earlier important findings of preferential LPC-DHA incorporation, as compared to NEFA-DHA, into the brain^19^,^20^,^22^. Similar to the reports of others^20^,^22^,^34^, our results suggest that upon a single intravenous bolus dose or infusion of LPC-DHA, more LPC-derived DHA will target the brain as compared to NEFA-DHA. This has therapeutic importance for studies aiming to deliver DHA to the brain and is consistent with preclinical findings regarding the efficacy of LPC-DHA analogues targeting the brain in models of stroke^35^. However, despite this “preferential LPC-DHA uptake”, LPC-DHA is not the major mechanism by which DHA enters the brain under normal conditions. LPC-DHA appears to circulate with a longer plasma half-life than NEFA-DHA. When controlling for plasma exposure and/or the steady-state non-labeled “cold” pool size, plasma NEFA-DHA is the major pool supplying the brain with DHA *in vivo*. It is important to note, that while the NEFA and LPC-DHA tracers disappear from the plasma with different half-lives, the cold concentrations remain at steady-state due to replacement from other pools. Based on results from experiments similar to ours (Fig. 2) and because mice lacking the candidate transporter for LPC-DHA Mfsd2a had deficits in brain DHA levels (~8% in phospholipids) compared to wild-type controls (~20%), Nguyen and colleagues proposed that the major mechanism by which DHA enters the brain is from plasma-derived LPC^22^. The large decrease in brain phospholipid DHA in Mfsd2a knockout mice may appear to be at odds with our finding that LPC-DHA is a relatively minor source of brain DHA, but there are many possible explanations for this apparent discrepancy, including: (a) the low DHA levels in the Mfsd2a knockout are due to the large loss of DHA-rich neuronal cells in the knockout (over 50% loss of Purkinje cells), possibly also explaining why DHA supplementation does not restore brain DHA levels or (b) that the Mfsd2a knockout was life-long and much brain DHA accretion occurs during development which may require LPC-DHA. Furthermore it is worth noting that despite life-long deficiency of Mfsd2a, knockout mice still have 8% DHA which is similar to what is reported during omega-3 deficiency in the mouse, albeit methodological differences may account for this result^36^,^37^.

The finding that plasma NEFA is the major plasma pool supplying the brain with DHA suggests that the previous studies that have estimated brain DHA uptake to be between 2.4 and 3.8 mg/brain/day, by imaging brain DHA uptake upon infusion of labeled NEFA-DHA in humans, are only slight underestimates of the total brain DHA uptake rate^7^,^38^,^39^. While it is known that the plasma NEFA pool is supplied by the adipose and circulating lipoproteins^40^, studies on the source of DHA in the plasma NEFA pool are, to the best of our knowledge, lacking. The DHA content in rat white adipose tissue was recently reported to be 13 mg^41^. Given our calculated rate of uptake of about 20 μg/day, this would suggest that adipose could supply for the brain for 1.8 years. However, this is an overestimate as DHA released from adipose does not solely supply to the brain and released DHA would be taken up by other tissues. Even though the brain/body partition coefficient for DHA has not been reported for rats, the partition coefficient has been estimated to be 5% for palmitate in rats and 0.5% for DHA in a monkey^23^,^39^. It is important to note that these might also be underestimates as fatty acids that are taken up by other tissues may be released and taken up by the brain at a later time. Also, a proportion, if not all, of the released DHA from adipose is being replaced from the diet^4^. Using similar calculations, we have recently estimated that adipose in adult humans contains enough DHA (20–50 g) to supply the brain for 14–36 years^42^.

In summary, our results demonstrate that the NEFA pool of DHA is the major source of DHA supplying the rat brain *in vivo* and confirm that a bolus of plasma LPC-DHA will target the brain more than a bolus of NEFA-DHA. Collectively, our results resolve a more than 20 year-old controversy and will inform studies aiming to deliver or image/quantify DHA entry into the brain which is being examined in stroke^43^,^44^, traumatic brain injury^8^,^45^ and other neurological diseases^12^,^38^,^46^,^47^. Future studies should examine the effects of diet, genetics, brain injury, disease, development and aging to test if the mechanisms presented here persist.

## Online Methods

All procedures were performed in accordance with the policies set out by the Canadian Council on Animal Care and were approved by the Animal Ethics Committee at the University of Toronto. Male Sprague Dawley rats were purchased from Charles Rivers (Saint-Constant, QC, Canada) at 10 weeks of age and kept at the animal facility with automated 12 hour light-dark cycle and a constant temperature of 22 °C for five weeks. The rats received *ad libitum* access to water and standard chow (Teklad 2018, Harlan, Madison, WI, USA) which was composed of 54% of linoleate (18:2n-6), 5% of α-linolenate (18:3n-3) and <0.3% of longer chained PUFA (20:2n-6, 20:3n-3, 20:4n-6, 20:5n-3, 22:4n-6, 22:5n-6, 22:5n-3 and DHA), as measured by gas chromatography-flame ionization detection (GC-FID).

### Radiotracer perfusate

Radiolabeled NEFA-^14^C-DHA ([1-^14^C]-DHA, specific activity: 58 mCi/mmol, Moravek Biochemical Inc., Brea, CA, USA) were solubilized in olive oil for gavage, and 5 mM HEPES buffer containing 50 mg/ml fatty acid-free bovine serum albumin for intravenous (iv) infusion (continuous and bolus) or 5 mM HEPES buffer containing 100 mg/ml fatty acid-free bovine serum albumin for intracerebroventricular (icv) infusion. The NEFA-^14^C-DHA perfusates were prepared to a concentration of 100 μCi/ml, 78 μCi/ml or 2 μCi/μl for the gavage, iv or icv study, respectively. The purity of ^14^C-radiotracer was >99.9% as confirmed by high performance liquid chromatography (HPLC) and liquid scintillation counting (LSC) (Supp Fig. 1). Radiolabeled LPC-^14^C-DHA (1-docosahexaenoyl-2-lysophosphocholine (Lysophosphatidylcholine, L-a-1-docosahexaenoyl [1-^14^C]), specific activity: 55 mCi/mmol, American Radiolabeled Chemicals Inc., St. Louis, MO, USA) were solubilized in 5 mM HEPES buffer containing 100 mg/ml fatty acid-free bovine serum albumin for iv infusions. The LPC-^14^C-DHA perfusates were prepared to a concentration of 33 μCi/ml or 8.6 μCi/ml for iv bolus and continuous infusion, respectively.

### Gavage

Four 15-week-old rats were anesthetised with isofluorane inhalation (3% induction, 1–2% maintenance). Rats were given a lower back subcutaneous injection of 100 mg/kg of Ketoprofen (MERIAL Canada, Inc., Baie d’Urfé, QC, Canada). Polyethylene catheters (PE 50, Intramedic™, Becton Dickinson, Franklin Lakes, NJ, USA) with silicone tubing (Silicone tubing 0.020 in (I.D.) and 0.037 in (O.D.), VWR®, Mississauga, ON, Canada) filled with 0.9% saline were implanted into the carotid artery. Surgery lasted for approximately 15 minutes. After surgery, all rats were singly housed to recover from anesthesia for 24 hours with *ad libitum* access to food and water. Rats were not fasted for the radiotracer oral gavage. Twenty-four hours post-surgery, rats were gavaged with 500 μCi of NEFA-^14^C-DHA (8.62 μmol DHA) in olive oil. Blood samples were obtained prior to gavage and at 15, 30, 45, 60, 90, 120, 180, and 240 minute minute post-gavage. After 4 hours, the rats were rapidly euthanized by head-focused, high-energy microwave irradiation (MI; 13.5 kW for 1.6 seconds; Cober Electronics Inc., Norwalk, CT, USA). Brains were excised and stored at −80 °C for radioactive and biochemical analyses. Plasma was isolated from whole blood via microcentrifugation at 6200 rpm (2000 g) for 10 minutes and stored at −80 °C.

### Intravenous (iv) continuous tracer infusion

Fourteen 15-week-old rats were anesthetised with isofluorane inhalation (3% induction, 1–2% maintenance). Rats were implanted with polyethylene catheters into the right carotid artery and right jugular vein that were buried in a skin pouch above the scapula and randomized to receive either an iv dose of 86 μCi NEFA-^14^C-DHA, 10 μCi NEFA-^14^C-DHA or 10 μCi LPC-^14^C-DHA which translated to 1.48 μmol, 0.17 μmol or 0.18 μmol of DHA over 5 minutes as previously described^25^.

### Intravenous (iv) bolus tracer infusion

Eight 15-week-old rats were implanted with catheters in the right carotid artery and right jugular vein which were externalized and protected by skin button (Braintree Scientific, Braintree, MA, USA) sutured to the subcutaneous tissue at the scapula, and hand-infused with 13 μCi of either NEFA-^14^C-DHA or LPC-^14^C-DHA which translated to 0.22 μmol or 0.24 μmol of DHA, respectively. Following hand-infusion of radiotracer, the catheter was immediately flushed with saline. Blood samples were collected at 0, 0.25, 0.5, 1, 5, 15, 30, 45, 60, 90, 120 minutes post-infusion. After two hours, the rats were rapidly euthanized by MI. Brains were excised and stored at −80 °C for radioactive and biochemical analyses. Plasma was isolated from whole blood via microcentrifugation and stored at −80 °C.

### Intracerebroventricular (icv) tracer infusion

At 15 weeks of age, eight rats were euthanized by MI for baseline brain fatty acid concentration measurements and seventeen 15-week-old rats were administered a 5 μl icv injection containing 10 μCi of ^14^C-DHA as previously described by Chen *et al*.^48^.

### Lipid analysis

Total lipids from whole brain (icv), one brain hemisphere (gavage and iv) and plasma (gavage and iv) were extracted and various neutral lipid and phospholipid classes from total lipid extract were isolated as previously described by Chen *et al*.^48^. Lysophosphatidylcholine (LPC) and lysophosphatidylethanolamine (LPE) from plasma were isolated by the method of Wang and Gustafson^49^. LPC and LPE were separated on TLC H-plates (Analtech, Newark, DE, USA) along with authentic standards. A day prior to sample loading, plates were washed in 0.4% ammonium sulphate. Plates were removed to air dry at room temperature for 2 hours and activated at 110 °C for 20 minutes prior to use. Samples were loaded onto 4 cm lanes. Plates were developed chloroform:methanol:acetic acid:acetone: water (40:25:7:4:2 by vol). Bands corresponding to authentic standards of LPC and LPE were visualized under UV light after spraying with 0.1% 8-anilino-1-naphthalene sulfonic acid and collected for liquid scintillation counting (LSC).

### NEFA-DHA and LPC-DHA analysis

Liquid chromatography (LC)/tandem mass spectrometry (MS/MS) was performed on an Agilent 1290 HPLC System (Agilent Technologies: Santa Clara, California, USA) and an ABSciex QTRAP5500 Mass Spectrometer (AB Sciex, Framingham, Massachusetts, USA). NEFA-DHA chromatography ran at a flow rate of 600 μL/min on an Agilent Zorbax SD-Phenyl 3.5 μ 3.0 × 50 mm column (Agilent Technologies). The mobile phase consisted of (A) water and (B) acetonitrile, both containing 1 μL propionic acid/250 mL solvent. The gradient was held at initial conditions of 20% B, holding for 2 minutes and ramping to 100% B at 8 minutes, held for 1 minute and returning to initial conditions. The mass spectrometer was operated in negative ESI mode with a source temperature of 600 °C, source voltage of −4500 V, curtain gas setting of 30, and collision gas N2 at medium. Mass transitions used for DHA were 327.15 to 283.15 m/z and for internal standard, DHA-d5 (Cayman Chemical, Ann Arbor, Michigan, USA), is 332.15 to 288.15 m/z. LPC-DHA chromatography ran at a flow rate of 800 μL/min on a Phenomenex Kinetex HILIC 2.6 μ 50 × 4.6 mm column (Phenomenex, Torrance, California, USA). The mobile phase consisted of (A) 90:10 water: acetonitrile and (B) 10:90 water: acetonitrile both containing 5 mM ammonium formate pH 3.2. The gradient was held at initial conditions of 10% A for 1 minute, ramping to 30% A at 3.5 minutes and returning to initial conditions. The mass spectrometer was operated in positive ESI mode with a source temperature of 600 °C, source voltage of +5500 V, curtain gas setting of 30, and collision gas N2 set at medium. Mass transitions used for LPC-DHA were 568.4 to 184.1 m/z and for internal standard, LPC-17:0 (Avanti Polar Lipids), were 510.4 to 184.1 m/z. Data integration and quantitation was performed using AB Sciex Analyst 1.6 software.

### Gas chromatography (GC)-flame ionization detection (FID)

Plasma NEFA concentration and brain fatty acid concentration at baseline were quantified as described by Chen *et al*.^48^.

### High performance liquid chromatography (HPLC)-UV photodiode array detection and liquid scintillation counting (LSC)

Fatty acid methyl esters (FAME) analysis were previously described by Chen *et al*.^48^.

### Matrix-assisted laser desorption/ionization (MALDI)-mass spectral imaging

The brain tissue block was mounted onto the specimen disc of a cryostat (Leica Microsystems) using optimal cutting temperature (OCT) compound (Sakura Finetek, Torrance, CA). The tissue sections were sliced at a thickness of 12 μm at −15 °C and mounted onto indium tin oxide (ITO)-coated glass slides. A thin matrix layer was applied to the tissue sections using an automated MALDI plate matrix deposition system (TM-Sprayer™, Leap Technologies, Carrboro, NC). A total of 5 ml of DHB solution (15 mg/ml in 50% acetonitrile/0.1% trifluoroacetic acid) or 9-aminoacridine (9AA, 15 mg/mL in methanol) was sprayed per slide during 4 passes at 140 °C (DHB) or at 80 °C (9AA) with a velocity of 400 mm/min and a line spacing of 3 mm. A time-of-flight tandem mass spectrometer (AB Sciex TOF/TOFTM 5800 System, AB SCIEX, Ontario, Canada) was used to acquire the images. MALDI-mass spectra were obtained using a Nd:YAG laser (349 nm) at 3 ns pulse width and 400 Hz firing rate. To install the ITO-coated glass slides in the ionization chamber, we used a special holder (AB Sciex) having concavities. The data were acquired in the positive-ion or negative-ion reflector mode using an external calibration method. The external calibration lipids (Avanti Polar Lipids) were deposited on the ITO-coated slides to minimize mass shift. In the imaging experiment, a total of 200 laser shots per point were irradiated (1s/point) and the interval between data points were 80 μm. The mass spectrometric data were processed using a specialized script of Analyst software (AB Sciex) at a mass resolution of 0.1 amu and images were visualized using TissueView software (AB Sciex). After MALDI imaging was completed, the glass coverslip containing the placenta section was carefully removed from the MALDI plate, dipped in methanol to remove the matrix and fixed prior to DAPI staining.

Slices were prepared with Vectashield (Vector Laboratories) mounting medium containing DAPI and visualized at 368nm on a widefield epi-fluorescent microscope at 4× magnification. Multiple images were stitched together to make full image using Nikon Elements Software (NIS-Elements Basic Research 3.10).

### Kinetics

To estimate the relative contribution of plasma NEFA (UE) and esterified (ES) DHA to brain phospholipids, the following equation were derived:


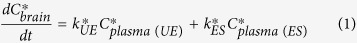


where C_*brain_ is the radioactivity in the brain (nCi/g brain) from DHA at time, *T*, k_*UE/ES_ is unidirectional incorporation coefficient (μl/s/g brain) which represents the incorporation of plasma ^14^C-DHA into stable brain lipid pools, and C_*plasma (UE/ES)_ is the radioactivity in the plasma (nCi/ml plasma) from DHA at time, *T*, of NEFA-^14^C-DHA gavaged rats. Integration of Equation 1 from the start of radiotracer infusion to time of death at *T* will yield:


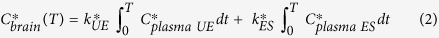


In order to calculate the relative contribution of esterified DHA to brain phospholipids, Equation 2 can be rearranged to solve for k_*ES_:


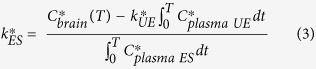


However, to calculate k_*ES_, k_*UE_ was separately measured from intravenous infusion of NEFA-^14^C-DHA where an acute five-minute infusion measures the uptake of albumin-bound NEFA-^14^C-DHA without ^14^C-DHA incorporation into phospholipids, triacylglycerol, cholesteryl esters, and diacylglycerol via hepatic lipoprotein packaging. k_*UE_ is often referred to as *k*.^*^


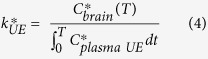


Since the incorporation coefficient applies to both radiolabeled and non-radiolabeled fatty acids, we can further calculate the rate of incorporation of non-radiolabeled plasma DHA into stable brain lipid pools, *J*_in_.


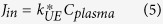


where *C*_plasma_ is the plasma NEFA-DHA concentration. In addition to the calculation of relative contribution of NEFA-DHA to brain phospholipid DHA uptake, the comparison of *J*_in_ from iv infusion and rate of loss, *J*_out_, from icv infusion can infer to the amount of plasma lipid pool(s) contributing to the maintenance of brain phospholipid DHA levels.

Loss half-lives, *t*_1/2_ (day), of ^14^C-DHA in brain total and phospholipid classes were calculated from the slope of regression lines (day^−1^) which were obtained by plotting log-transformed ^14^C-DHA radioactivity against day post-icv infusion.


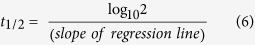


The *t*_1/2_ of DHA was used to calculate the *J*_out_ (nmol/g brain/day) from brain phospholipids by the following equation:


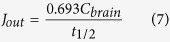


where *C*_*brain*_ is the baseline DHA concentration in a stable phospholipid pool (nmol/g brain). If the NEFA-DHA pool is the major contributor to total brain DHA uptake, then *J*_in_ would equal *J*_out_. By rearranging Equation 5, predictive k*_UE_ can be solved:


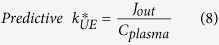


Finally, to calculate a fractional loss (%/day) of DHA from stable phospholipid pool.


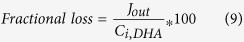


### Statistics

Concentrations and kinetic parameters are expressed as mean ± SEM. HPLC profiles were analyzed as pooled samples and do not have SEM. Statistical comparisons of gavage k_*UE_/*J*_in_ and iv k_*UE_/*J*_in_ as well as icv predictive k_*UE_ and gavage/iv k_*UE_ were performed, *a priori*, using two tailed Student’s *t*-test. Statistical significant differences were denoted as ^*^p < 0.05, ^**^p < 0.01 and ^***^p < 0.001.

## Additional Information

**How to cite this article**: Chen, C. T. *et al*. Plasma non-esterified docosahexaenoic acid is the major pool supplying the brain. *Sci. Rep*. **5**, 15791; doi: 10.1038/srep15791 (2015).

## Supplementary Material

Supplementary Information

## References

[b1] Diau G. Y. . The influence of long chain polyunsaturate supplementation on docosahexaenoic acid and arachidonic acid in baboon neonate central nervous system. BMC Med 3, 11, doi: 10.1186/1741-7015-3-11 (2005). 15975147 PMC1184078

[b2] Serhan C. N. Pro-resolving lipid mediators are leads for resolution physiology. Nature 510, 92–101, doi: 10.1038/nature13479 (2014). 24899309 PMC4263681

[b3] Kim H. Y., Spector A. A. & Xiong Z. M. A synaptogenic amide N-docosahexaenoylethanolamide promotes hippocampal development. Prostaglandins Other Lipid Mediat 96, 114–120, doi: 10.1016/j.prostaglandins.2011.07.002S1098-8823(11)00063-3 (2011). 21810478 PMC3215906

[b4] Bazinet R. P. & Laye S. Polyunsaturated fatty acids and their metabolites in brain function and disease. Nat Rev Neurosci 15, 771–785, doi: 10.1038/nrn3820nrn3820 (2014). 25387473

[b5] Kim H. Y. Novel metabolism of docosahexaenoic acid in neural cells. J Biol Chem 282, 18661–18665, doi: R70001520010.1074/jbc.R700015200 (2007). 17488715 10.1074/jbc.R700015200

[b6] Liu J. J., Green P., John Mann J., Rapoport S. I. & Sublette M. E. Pathways of polyunsaturated fatty acid utilization: Implications for brain function in neuropsychiatric health and disease. Brain research 1597C, 220–246, doi: S0006-8993(14)01666-710.1016/j.brainres.2014.11.059 (2015). 25498862 10.1016/j.brainres.2014.11.059 PMC4339314

[b7] Cao D. . Docosahexaenoic acid promotes hippocampal neuronal development and synaptic function. J Neurochem 111, 510–521, doi: 10.1111/j.1471-4159.2009.06335.xJNC6335 (2009). 19682204 PMC2773444

[b8] Kim H. Y. Neuroprotection by docosahexaenoic acid in brain injury. Military medicine 179, 106–111, doi: 10.7205/MILMED-D-14-00162 (2014). 25373093

[b9] Calon F. . Docosahexaenoic acid protects from dendritic pathology in an Alzheimer’s disease mouse model. Neuron 43, 633–645, doi: 10.1016/j.neuron.2004.08.013S0896627304005227 (2004). 15339646 PMC2442162

[b10] Calon F. Omega-3 polyunsaturated fatty acids in Alzheimer’s disease: key questions and partial answers. Curr Alzheimer Res 8, 470–478, doi: BSP/CAR/0173 (2011). 21605051 10.2174/156720511796391881

[b11] Palacios-Pelaez R., Lukiw W. J. & Bazan N. G. Omega-3 essential fatty acids modulate initiation and progression of neurodegenerative disease. Mol Neurobiol 41, 367–374, doi: 10.1007/s12035-010-8139-z (2010). 20467837

[b12] Salem N. Jr., Vandal M. & Calon F. The benefit of docosahexaenoic acid for the adult brain in aging and dementia. Prostaglandins, leukotrienes, and essential fatty acids 92, 15–22, doi: 10.1016/j.plefa.2014.10.003 (2015). 25457546

[b13] Rapoport S. I., Rao J. S. & Igarashi M. Brain metabolism of nutritionally essential polyunsaturated fatty acids depends on both the diet and the liver. Prostaglandins Leukot Essent Fatty Acids 77, 251–261, doi: S0952-3278(07)00155-X10.1016/j.plefa.2007.10.023 (2007). 18060754 10.1016/j.plefa.2007.10.023 PMC2725409

[b14] Scott B. L. & Bazan N. G. Membrane docosahexaenoate is supplied to the developing brain and retina by the liver. Proceedings of the National Academy of Sciences of the United States of America 86, 2903–2907 (1989). 2523075 10.1073/pnas.86.8.2903 PMC287028

[b15] DeMar J. C. Jr., Ma K., Chang L., Bell J. M. & Rapoport S. I. alpha-Linolenic acid does not contribute appreciably to docosahexaenoic acid within brain phospholipids of adult rats fed a diet enriched in docosahexaenoic acid. J Neurochem 94, 1063–1076 (2005). 16092947 10.1111/j.1471-4159.2005.03258.x

[b16] DeMar J. C. Jr., Ma K., Bell J. M. & Rapoport S. I. Half-lives of docosahexaenoic acid in rat brain phospholipids are prolonged by 15 weeks of nutritional deprivation of n-3 polyunsaturated fatty acids. Journal of neurochemistry 91, 1125–1137, doi: 10.1111/j.1471-4159.2004.02789.x (2004). 15569256

[b17] Chen C. T. & Bazinet R. P. beta-oxidation and rapid metabolism, but not uptake regulate brain eicosapentaenoic acid levels. Prostaglandins, leukotrienes, and essential fatty acids 92, 33–40, doi: 10.1016/j.plefa.2014.05.007 (2015). 24986271

[b18] Dietschy J. M. Central nervous system: cholesterol turnover, brain development and neurodegeneration. Biol Chem 390, 287–293, doi: 10.1515/BC.2009.035 (2009). 19166320 PMC3066069

[b19] Thies F., Pillon C., Moliere P., Lagarde M. & Lecerf J. Preferential incorporation of sn-2 lysoPC DHA over unesterified DHA in the young rat brain. The American journal of physiology 267, R1273–1279 (1994). 7977854 10.1152/ajpregu.1994.267.5.R1273

[b20] Thies F., Delachambre M. C., Bentejac M., Lagarde M. & Lecerf J. Unsaturated fatty acids esterified in 2-acyl-l-lysophosphatidylcholine bound to albumin are more efficiently taken up by the young rat brain than the unesterified form. J Neurochem 59, 1110–1116 (1992). 1494901 10.1111/j.1471-4159.1992.tb08353.x

[b21] Green P. & Yavin E. Mechanisms of docosahexaenoic acid accretion in the fetal brain. J Neurosci Res 52, 129–136, doi: 10.1002/(SICI)1097-4547(19980415)52:2<129::AID-JNR1>3.0.CO;2-C(1998 ). 9579403

[b22] Nguyen L. N. . Mfsd2a is a transporter for the essential omega-3 fatty acid docosahexaenoic acid. Nature 509, 503–506, doi: 10.1038/nature13241 (2014). 24828044

[b23] Robinson P. J. . A quantitative method for measuring regional *in vivo* fatty-acid incorporation into and turnover within brain phospholipids: review and critical analysis. Brain Res Brain Res Rev 17, 187–214 (1992). 1467810 10.1016/0165-0173(92)90016-f

[b24] Purdon D., Arai T. & Rapoport S. No evidence for direct incorporation of esterified palmitic acid from plasma into brain lipids of awake adult rat. J Lipid Res 38, 526–530 (1997). 9101433

[b25] Chen C. T. . The low levels of eicosapentaenoic acid in rat brain phospholipids are maintained via multiple redundant mechanisms. J Lipid Res 54, 2410–2422, doi: 10.1194/jlr.M038505 (2013). 23836105 PMC3735939

[b26] Lin L. E. . Chronic dietary n-6 PUFA deprivation leads to conservation of arachidonic acid and more rapid loss of DHA in rat brain phospholipids. Journal of lipid research 56, 390–402, doi: 10.1194/jlr.M055590 (2015). 25477531 PMC4306692

[b27] Croset M., Brossard N., Polette A. & Lagarde M. Characterization of plasma unsaturated lysophosphatidylcholines in human and rat. Biochem J 345 Pt 1, 61–67 (2000). 10600639 PMC1220730

[b28] Rapoport S. I., Chang M. C. & Spector A. A. Delivery and turnover of plasma-derived essential PUFAs in mammalian brain. Journal of lipid research 42, 678–685 (2001). 11352974

[b29] Kamp F. & Hamilton J. A. pH gradients across phospholipid membranes caused by fast flip-flop of un-ionized fatty acids. Proceedings of the National Academy of Sciences of the United States of America 89, 11367–11370 (1992). 1454821 10.1073/pnas.89.23.11367 PMC50551

[b30] Kamp F., Zakim D., Zhang F., Noy N. & Hamilton J. A. Fatty acid flip-flop in phospholipid bilayers is extremely fast. Biochemistry 34, 11928–11937 (1995). 7547929 10.1021/bi00037a034

[b31] Ouellet M. . Diffusion of docosahexaenoic and eicosapentaenoic acids through the blood-brain barrier: An *in situ* cerebral perfusion study. Neurochemistry international 55, 476–482 (2009). 19442696 10.1016/j.neuint.2009.04.018

[b32] Kamp F. & Hamilton J. A. How fatty acids of different chain length enter and leave cells by free diffusion. Prostaglandins Leukot Essent Fatty Acids 75, 149–159, doi: S0952-3278(06)00081-010.1016/j.plefa.2006.05.003 (2006). 16829065 10.1016/j.plefa.2006.05.003

[b33] Hamilton J. A., Guo W. & Kamp F. Mechanism of cellular uptake of long-chain fatty acids: Do we need cellular proteins? Molecular and cellular biochemistry 239, 17–23 (2002). 12479564

[b34] Lemaitre-Delaunay D. . Blood compartmental metabolism of docosahexaenoic acid (DHA) in humans after ingestion of a single dose of [(13)C]DHA in phosphatidylcholine. J Lipid Res 40, 1867–1874 (1999). 10508206

[b35] Lagarde M. . Biological properties of a DHA-containing structured phospholipid (AceDoPC) to target the brain. Prostaglandins Leukot Essent Fatty Acids 92, 63–65, doi: 10.1016/j.plefa.2014.01.005S0952-3278(14)00019-2 (2015). 24582148

[b36] Hussein N. . Artificial rearing of infant mice leads to n-3 fatty acid deficiency in cardiac, neural and peripheral tissues. Lipids 44, 685–702, doi: 10.1007/s11745-009-3318-2 (2009). 19588181 PMC2771777

[b37] Lafourcade M. . Nutritional omega-3 deficiency abolishes endocannabinoid-mediated neuronal functions. Nat Neurosci 14, 345–350, doi: 10.1038/nn.2736 (2011). 21278728

[b38] Umhau J. C. . Brain docosahexaenoic acid [DHA] incorporation and blood flow are increased in chronic alcoholics: a positron emission tomography study corrected for cerebral atrophy. PloS one 8, e75333, doi: 10.1371/journal.pone.0075333 (2013). 24098376 PMC3788756

[b39] Umhau J. C. . Imaging incorporation of circulating docosahexaenoic acid into the human brain using positron emission tomography. J Lipid Res 50, 1259–1268, doi: 10.1194/jlr.M800530-JLR200 (2009). 19112173 PMC2694326

[b40] Frayn K. N., Summers L. K. & Fielding B. A. Regulation of the plasma non-esterified fatty acid concentration in the postprandial state. Proc Nutr Soc 56, 713–721, doi: S0029665197000244 (1997). 9264121 10.1079/pns19970071

[b41] Salem N. M. . Distribution of omega-6 and omega-3 polyunsaturated fatty acids in the whole rat body and 25 compartments. Prostaglandins Leukot Essent Fatty Acids, doi: S0952-3278(15)00122-210.1016/j.plefa.2015.06.002 (2015). 10.1016/j.plefa.2015.06.002 PMC4555191 26120061

[b42] Domenichiello A. F., Kitson A. P. & Bazinet R. P. Is docosahexaenoic acid synthesis from alpha-linolenic acid sufficient to supply the adult brain? Progress in lipid research 59, 54–66, doi: 10.1016/j.plipres.2015.04.002S0163-7827(15)00022-3 (2015). 25920364

[b43] Hong S. H., Belayev L., Khoutorova L., Obenaus A. & Bazan N. G. Docosahexaenoic acid confers enduring neuroprotection in experimental stroke. J Neurol Sci 338, 135–141, doi: 10.1016/j.jns.2013.12.033S0022-510X(13)03104-3 (2014). 24433927 PMC3943637

[b44] Eady T. N. . Docosahexaenoic acid complexed to albumin provides neuroprotection after experimental stroke in aged rats. Neurobiol Dis 62, 1–7, doi: 10.1016/j.nbd.2013.09.008S0969-9961(13)00254-4 (2014). 24063996 PMC3877728

[b45] Barrett E. C., McBurney M. I. & Ciappio E. D. omega-3 fatty acid supplementation as a potential therapeutic aid for the recovery from mild traumatic brain injury/concussion. Advances in nutrition 5, 268–277, doi: 10.3945/an.113.005280 (2014). 24829473 PMC4013179

[b46] Vandal M. . Reduction in DHA transport to the brain of mice expressing human APOE4 compared to APOE2. Journal of neurochemistry 129, 516–526, doi: 10.1111/jnc.12640 (2014). 24345162

[b47] Taha A. Y., Burnham W. M. & Auvin S. Polyunsaturated fatty acids and epilepsy. Epilepsia 51, 1348–1358, doi: 10.1111/j.1528-1167.2010.02654.x (2010). 20608961

[b48] Chen C. T., Liu Z. & Bazinet R. P. Rapid de-esterification and loss of eicosapentaenoic acid from rat brain phospholipids: an intracerebroventricular study. J Neurochem 116, 363–373, doi: 10.1111/j.1471-4159.2010.07116.x (2011). 21091476

[b49] Wang W. Q. & Gustafson A. One-dimensional thin-layer chromatographic separation of phospholipids and lysophospholipids from tissue lipid extracts. J Chromatogr 581, 139–142 (1992). 1429997 10.1016/0378-4347(92)80457-2

