# Supplementary material for: Plasma non-esterified docosahexaenoic acid is the major pool supplying the brain
Source: Sci Rep. 2015 Oct 29;5:15791. doi: 10.1038/srep15791 (PMC4625162; doi:10.1038/srep15791)
Supplement: Supplementary Information [file srep15791-s1.pdf]

SUPPLEMENTARY FIGURES AND TABLE

Plasma non-esterified docosahexaenoic acid is the major pool supplying the brain.

Chuck T. Chen[1], Alex P. Kitson[1], Kathryn E. Hopperton[1], Anthony F. Domenichiello[1], Marc-Olivier Trépanier[1], Lauren E. Lin[1], Leonardo Ermini[2], Martin Post[2], Frank Thies[3] and Richard P. Bazinet[1]

Supplementary Table 1

	Gavage	iv	Gavage	iv
	k_{UE}^* (µl/s/g)		J_{in} (nmol/g/day)	
Total PL	0.098 ± 0.02	0.20 ± 0.02	32 ± 5	80 ± 10
ChoGpl	0.033 ± 0.008	0.054 ± 0.005	11 ± 2	21 ± 2
EtnGpl	0.045 ± 0.01	0.096 ± 0.01	14 ± 2	38 ± 6
PtdSer	0.0064 ± 0.002	0.0055 ± 0.001	2.0 ± 0.4	2.2 ± 0.4
PtdIns	0.0051 ± 0.001	0.036 ± 0.003	1.6 ± 0.2	14 ± 1.8

	icv			
	Predictive k_{UE}^* (µl/s/g)	$t_{1/2}$ (day)	J_{out} (nmol/g/day)	Fractional Loss (%/day)
Total PL	0.17 ± 0.02	64 ± 7	65 ± 7	1.1 ± 0.12
ChoGpl	0.030 ± 0.002	51 ± 4	12 ± 0.9	1.4 ± 0.11
EtnGpl	0.15 ± 0.01	57 ± 5	59 ± 5	1.2 ± 0.11
PtdSer	0.041 ± 0.007	75 ± 12	16 ± 2	0.93 ± 0.15
PtdIns	0.0026 ± 0.0002	53 ± 5	0.98 ± 0.09	1.3 ± 0.11

Supplementary Table 1. Uptake incorporation coefficient (k_{UE}^*), rate of incorporation (J_{in}) of NEFA-DHA from plasma to brain phospholipids and DHA loss kinetic parameters in rat brain phospholipids. Data from gavage and iv infusions are mean ± SEM (n = 4-6); while data from icv infusions are mean ± SEM (n = 3-4). Half-lives of ^{14}C-DHA were derived from the slopes using $t_{1/2} = \log_{10}2$/(slope of regression line). Baseline DHA concentrations were used to derive rate of loss (J_{out}) from brain lipid classes using $J_{out} = 0.693C_{brain\ DHA}/t_{1/2}$, where $C_{brain\ DHA}$ is the baseline brain DHA concentration. Total PL, total phospholipids; ChoGpl, choline glycerophospholipid; EtnGpl, ethanolamine glycerophospholipid; PtdSer, phosphatidylserine; PtdIns, phosphatidylinositol.

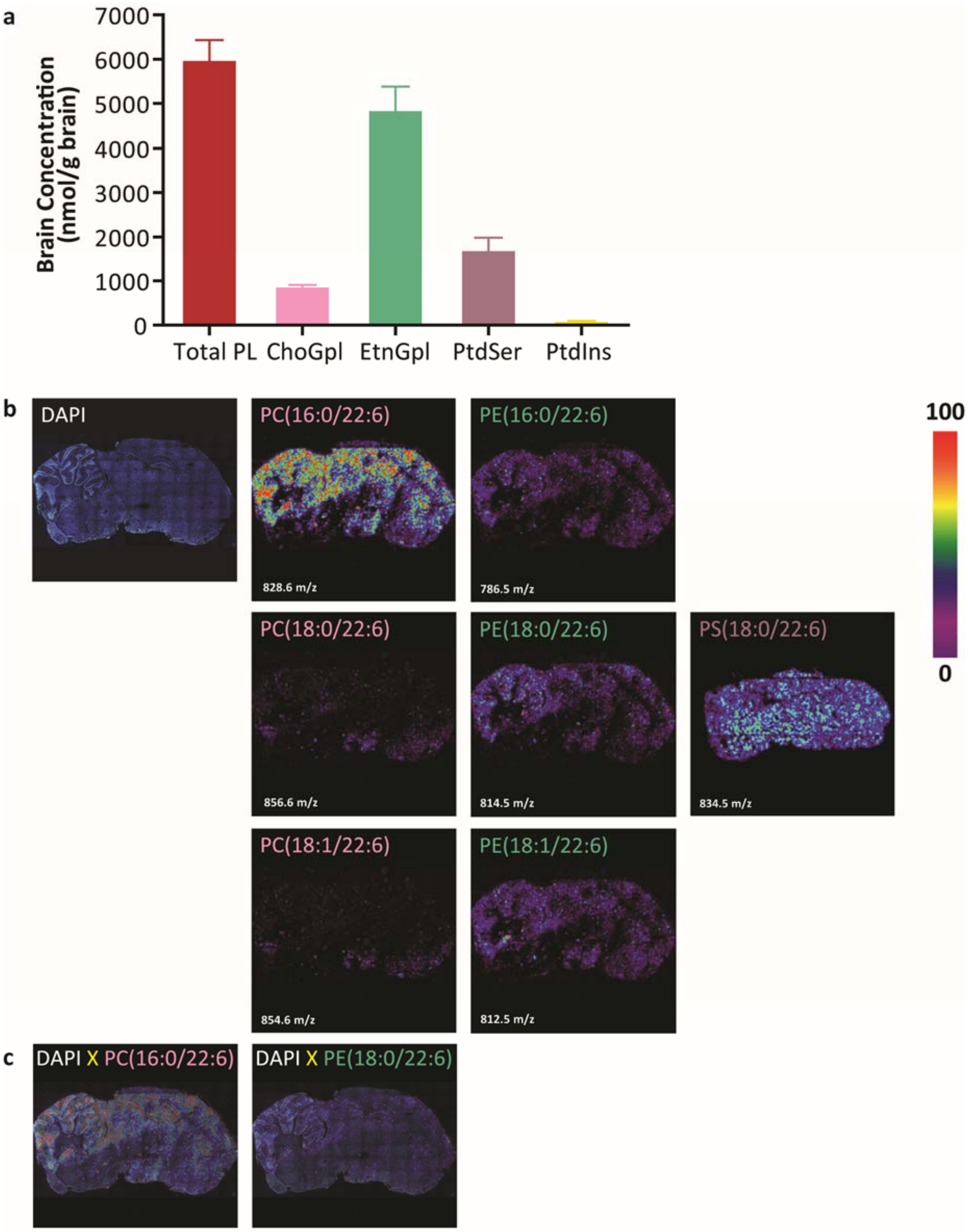

Supplementary Figure 1. Brain docosahexaenoic acid (DHA) concentration (**a**) and relative distribution (**b-c**). (**a**) DHA concentration in brain total phospholipids and four major phospholipid classes, including choline glycerophospholipids (ChoGpl), ethanolamine glycerophospholipids (EtnGpl), phosphatidylserine (PtdSer) and phosphatidylinositol (PtdIns) (n = 8). (**b**) Sagittal slice of microwave brain stained with DAPI or imaged with MALDI-mass

spectral imaging for various DHA-containing phosphatidylcholine (PC), phosphatidylethanolamine (PE) and phosphatidylserine (PS) species in negative-ion reflector mode. Intensities of the ions are represented in color based on the intensity scale. (**c**) Overlay of DAPI and MALDI images.

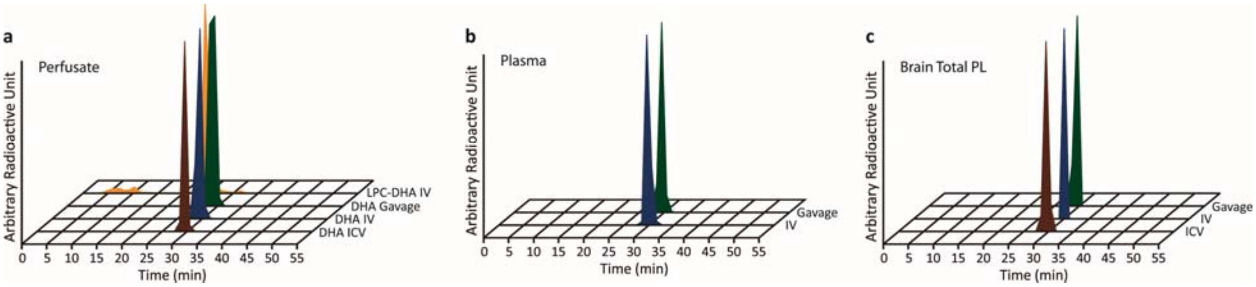

Supplementary Figure 2. HPLC separation of radioactivity in perfusate, total lipids in plasma and total phospholipids in brain. Peak identity was confirmed by authentic standards and GC-FID.

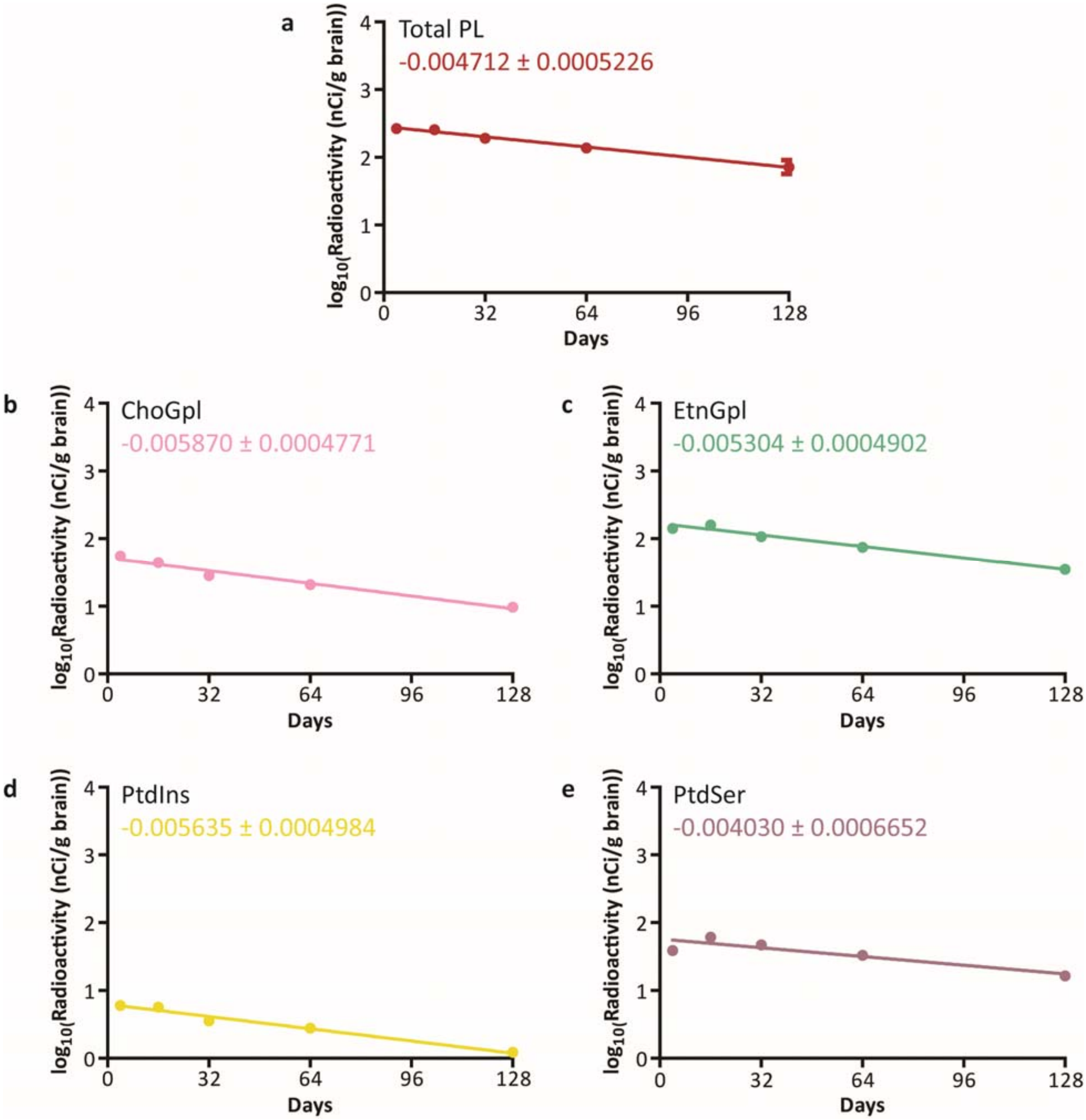

Supplementary Figure 3. Linear regression of $\log_{10}$Radioactivity (nCi/g brain) and days post-icv-infusion. Slopes, indicated by the best-fit line, were significantly difference from zero ($P < 0.05$). Data are expressed as mean ± SEM (n = 3-4 independent samples per time point). ChoGpl, choline glycerophospholipids; EtnGpl, ethanolamine glycerophospholipids; PtdIns, phosphatidylinositol; PtdSer, phosphatidylserine.
